# The Future of Beta Cells Replacement in the Era of Regenerative Medicine and Organ Bioengineering

**DOI:** 10.3389/ti.2024.12885

**Published:** 2024-03-13

**Authors:** Ekaterine Berishvili, Andrea Peloso, Alice A. Tomei, Andrew R. Pepper

**Affiliations:** ^1^ Laboratory of Tissue Engineering and Organ Regeneration, Department of Surgery, University of Geneva, Geneva, Switzerland; ^2^ Cell Isolation and Transplantation Center, Department of Surgery, Geneva University Hospitals and University of Geneva, Geneva, Switzerland; ^3^ Faculty Diabetes Center, University of Geneva Medical Center, Geneva, Switzerland; ^4^ Institute of Medical and Public Health Research, Ilia State University, Tbilisi, Georgia; ^5^ Transplantation and Visceral Surgery Division, University Hospitals of Geneva, Geneva, Switzerland; ^6^ Department of Biomedical Engineering, University of Miami, Miami, FL, United States; ^7^ Diabetes Research Institute, University of Miami Miller School of Medicine, Miami, FL, United States; ^8^ Department of Surgery, University of Alberta, Edmonton, AB, Canada; ^9^ Alberta Diabetes Institute, University of Alberta, Edmonton, AB, Canada

**Keywords:** islet transplantation, islets-on-chip, bioengineering, islet organoids, stem cell derived beta cells

As we move into 21st century, the landscape of type 1 diabetes (T1D) management is undergoing a significant transformation. Islet transplantation, initially hailed as a breakthrough in replacing lost insulin-producing beta cells, is now confronted with challenges such as the scarcity of donor pancreata, difficulties in predicting and ensuring successful islet engraftment, graft attrition and the need for chronic immunosuppression, all of which hinder its long-term efficacy and sustainability [1, 2].

To address these impediments the scientific community has made significant strides in various domains, including stem cell technology, xenotransplantation, encapsulation techniques and immunomodulatory strategies [3–7]. This progress is further amplified by advancements in tissue engineering strategies, including the generation of on-chip technologies and biomimetic scaffolds, the development of organoids containing both therapeutic and support cells, driving the bioengineering of the endocrine pancreas [7–11]. Concomitantly, the availability of long-term clinical islet transplantation data from different regions allows mapping the activity at the worldwide level to identify regional differences, developing and validating clinical scores to correlate early graft function with long-term transplant outcomes, and the inclusion of patient perspectives and wellbeing in future clinical implementation of current research.

This Special Issue showcases the forefront of beta-cell replacement research and clinical implantation, drawing together 14 rigorously peer-reviewed articles. Each paper highlights a different, yet interrelated aspect of islet transplantation, and collectively provides an in-depth analysis of the field’s current achievements and outline its future disruptive perspectives.

Bridging these comprehensive insights, the study by Lam et al. emerges as a significant highlight within this issue, introducing the BETA-2 score, as a new benchmark in predicting long-term transplant outcomes. This new approach sets a higher standard for the evaluation of transplantation procedures, by underscoring the importance of early and continued assessment of graft function, with the possibility of proactive intervention.

van de Leemkolk have provided a significant contribution with their novel technique for assessing β-cell damage in cultured islets by quantifying unmethylated insulin DNA by PCR. Their technique, based on methylation-sensitive restriction enzyme digital PCR, allows for the evaluation of the islet preparation’s purity and quality before transplantation, thus providing a noteworthy improvement in determining their viability and subsequent successful transplantation.

Further exploring the vital aspects of transplantation, Chetboun et al. investigate the correlation between primary graft function (PGF) and 5-year insulin independence in islet and pancreas transplant patients. Using the Beta-2 score for PGF assessment, their study shows a significant positive correlation between early PGF and long-term insulin independence in patients with T1D receiving either islet or pancreas transplantation, underscoring PGF’s predictive value in long-term transplantation outcomes.

In their study, Bond et al. analyze the relationship between islet graft function and wellbeing in islet transplant recipients with T1D. This study clearly demonstrates that despite some clinical benefits, “marginal” graft function is associated with suboptimal wellbeing, thus raising the potential need for additional interventions such as re-transplantation.

Berney et al.’s study on international collaboration in islet transplantation highlights the essential role of standardized practices in a field characterized by diverse methodologies and regulatory acceptance. Their findings set the tone for global cooperation and standardization in advancing T1D treatments.

Raoux et al.’s “Islets-on-chip” study introduces a novel approach to predicting clinical islet transplant outcomes by developing a CHIP-score based on donor islets’ electrical activity. This method, despite its early stage, could significantly enhance the evaluation and selection process of beta cell replacement therapies before transplantation and potentially predict their outcomes once infused.

Pignatelli et al. discuss the bioengineering of the vascularized endocrine pancreas which refers to pancreatic tissue bioengineered with a focus on the recapitulating its’ endogenous vascular structure and cytoarchitecture. These building-blocks are crucial to ensure that the tissue remains functional and can integrate, once transplanted, into the circulatory system with the immediate restoration of blood flow to supply nutrients and oxygen and remove waste products. In this scenario, the authors shed light on the intricate interplay of various crucial elements necessary for successful beta cell replacement including vascularization and extracellular matrix. This report provides guidance for the future development of more effective and sustainable beta cell replacement modalities.

Wassmer et al. investigate the development of pre-vascularized islet organoids, combining therapeutic islet cells with support cells (amniotic epithelial cells and endothelial cells). Their study shows how these organoids outperform native islets *in vitro* and demonstrate improved engraftment and vascularization *in vivo*, in a murine model of T1D. This advancement, attributed to enhanced cell-cell interaction and mediated by upregulation of both pro-angiogenic and pro-β-cell survival genes, suggests a promising approach for beta replacement therapies, hypothetically enabling transplantation in more favorable extrahepatic sites.

In their review, Sackett et al. discuss the potential of genome editing for the development of immune-evasive stem cell-derived islets, a breakthrough with significant implications for advancing transplantation medicine, broadening patient inclusion and reducing procedural risks by limiting the need for chronic systemic immunosuppression.

Pellegrini et al. address a paramount concern in the field: the safety of iPSC-derived beta cells. As more of these therapies approach clinical application, their roadmap for addressing safety concerns is invaluable and can be done through four different strategies, such as somatic cell reprogramming, purification of differentiated beta cells, depletion of contaminant stem cells, and reducing the risk of tumorigenicity through suicide genes.

To translate potential surrogates for human cadaveric donors, Honarpisheh et al. study introduces an innovative approach to re-aggregate dispersed neonatal porcine islet-like cell clusters (NPICCs) as an alternative source transplantable insulin-producing cells for the management of T1D. Their research demonstrates how the re-aggregated NPICCs (REPIs) form uniform clusters with enhanced functionality and *in vivo* performance. This significant finding suggests that re-aggregated NPICCs could expand the potential donor pool for islet xenotransplantation, with improved functionality and outcomes for clinical applications.

Tol et al.’s study, surveying over 800 patients with T1D and caregivers, reveals a high willingness (97%) to receive islet delivery devices (IDDs). The study also highlights patient flexibility regarding IDD characteristics, with device functionality duration outweighing size and number of implants required, underscoring the importance of patient-centered design in future beta cell replacement strategies.

de Jongh et al. address complex ethical, legal, and psychosocial considerations in bio-artificial pancreas therapies. Their advocacy for an interdisciplinary approach that includes patient perspectives is crucial in ensuring the ethical development of these therapies.

Lastly, Piemonti et al. discuss the potential of Advanced Therapy Medicinal Products (ATMPs) in transplantation. Their emphasis on the need for enhanced funding and streamlined regulatory processes to overcome current development bottlenecks is critical for the advancement and accessibility of these therapies.

Despite heightened awareness, the multifaceted nature of diabetes continues to present a complex challenge to patients, as well as to the clinicians, basic scientists and healthcare professionals responsible for diagnosing, researching, monitoring and managing it. Beta-cell replacement is currently recognized as the only definitive therapeutic intervention that can free patients with diabetes from the need to administer insulin externally thus improving survival rates and quality of life. As witnessed by this Special Issue, regenerative medicine and organ bioengineering are undergoing a surge of dynamic advances into new horizons and the treatment of T1D.

Herein we not only showcase groundbreaking research but also illuminate the path toward a future where beta cell replacement therapies are a mainstream, safe, durable, effective, and equitable treatment option for T1D. Each contribution in this issue advances our scientific understanding on beta cell replacement and challenges us to consider the broader implications of these innovations. This compilation serves as an invaluable resource for researchers, clinicians, and patients alike, paving the way for new discoveries and applications in the field of T1D management.
